# Small-area deprivation measure datasets for Scotland, 2001 and 2011

**DOI:** 10.1016/j.dib.2016.04.060

**Published:** 2016-04-30

**Authors:** Mirjam Allik, Denise Brown, Ruth Dundas, Alastair H Leyland

**Affiliations:** University of Glasgow, United Kingdom

**Keywords:** Deprivation measures, Social epidemiology, Standardized health data, Carstairs score, Scottish Index of Multiple Deprivation

## Abstract

These data present a new small-area deprivation measure, but also include a variety of other indicators, such as the Scottish Index of Multiple Deprivation (SIMD) and the Carstairs score. The data are for Scottish 2001 Datazones and for the years 2001 and 2011. In addition the data provide standardised self-reported measures of general health and limiting long-term illness. The theoretical background for developing the new deprivation measure, and the implications of using different measures to study health inequalities are discussed in “*Developing a new small-area measure of deprivation using 2001 and 2011 census data from Scotland*” (Allik et al., 2016) [1].

**Specifications Table**

**Table:** 

Subject area	*Social epidemiology*
More specific subject area	*Small-area deprivation measures*
Type of data	*Tables*
How data was acquired	*Publically available Scottish census data, commissioned census tables, Scottish Government data, data published by other researchers*
Data format	*Compiled datasets (.csv files)*
Experimental factors	*Data was compiled from multiple sources.*
Experimental features	*New indicators were calculated based on a combination of different data sources.*
Data source location	*Scotland*
Data accessibility	*Data are within this article.*

**Value of the data**

• The data includes a new deprivation measure developed from the Scottish census data.
• For the first time, the 2001 Carstairs scores are provided for Datazones.
• The data includes standardized self-reported health measures calculated from the Scottish census that can be used beyond health research, e.g. labor market or social care research.
• Deprivation measures can be used to study health inequalities, but also to study educational attainment, school attendance or provision of local services, such as housing or social care.

## 1 Data

These data provide a variety of single-variable deprivation indicators and three composite deprivation measures for 2001 Scottish Datazones for the census years 2001 and 2011. Standardized health measures and detailed population data are also provided. The data are in Supplementary Tables 1 “deprivation_measure_data_2001_dz.csv” and 2 “deprivation_measure_data_2001_dz.csv”. Supplementary Table 3 “deprivation_measure_data_dictionary.csv” provides a data dictionary.

## 2 Experimental design, materials and methods

The data are provided for all 6505 Scottish Datazones for 2001; for 2011 data is provided for 6500 Datazones (five had no population in 2011). Most of the data for the deprivation variables are from the 2001 and 2011 Scottish census [2]; the data for the SIMD and the Urban Rural classification are from the Scottish Government [3], [4], [5], [6]. The 2011 Carstairs scores are from Brown et al. [7] and the overcrowding variable for 2001 from Richardson [8]. Lists of the specific tables used to create the data set are provided in Table 1, Table 2. All tables listed in Table 2 and all but two tables listed in Table 1 are publically available.

The single-variable deprivation indicators included are: the percent of people with no educational qualifications, percent of people in socially rented accommodation, percent in overcrowded households, percent unemployed, percent unemployed men, percent of people in households where the household reference person (HRP) is in NS-SeC analytic classes 6 or 7, percent of people in households where the HRP is of low social class, and the percent of people in households with no access to a car or a van. The percent of people with no educational qualifications is age and gender standardised using Scottish population at the census year.

The deprivation measures include the new deprivation measure, SIMD, the SIMD income domain, and the Carstairs score. The new deprivation measure combines the percent of people with no educational qualifications, percent of people in socially rented accommodations, percent unemployed and percent of people in households where the HRP is in NS-SeC analytic classes 6 or 7. The Carstairs deprivation score excluding overcrowding is also provided.

**Fig. 1:**
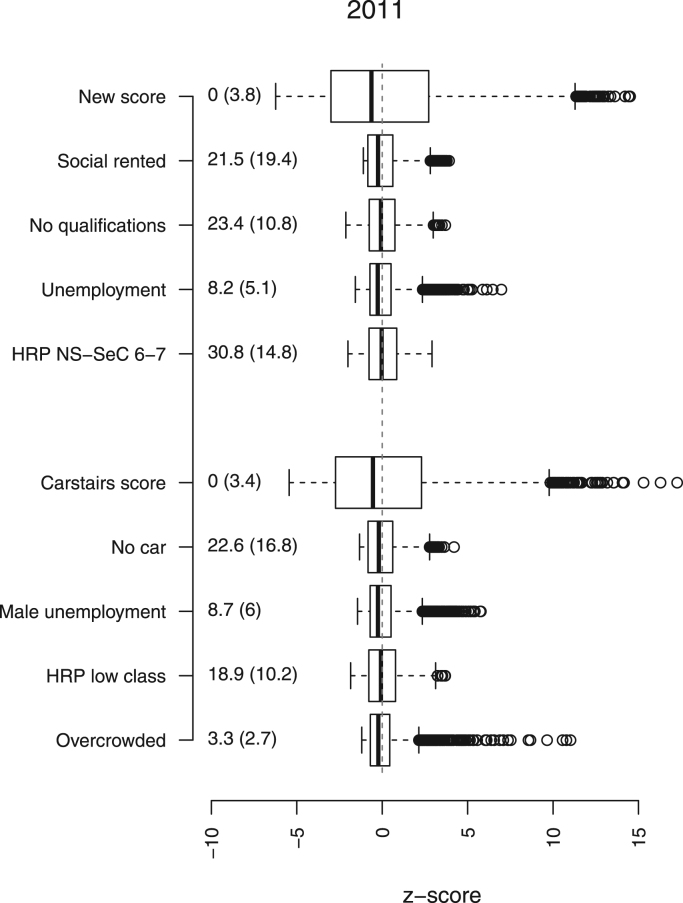
Distribution of deprivation scores and variables, 2011.

To calculate the new deprivation measure and the Carstairs score for 2001, we followed the *z*-score method described in Brown et al. [7]. We used household population to calculate the weighted means and standard deviations for the *z*-scores. Fig. 1 shows the population weighted means, standard deviations of the deprivation measures and the distributions of the *z*-scores in 2011. Positive values indicate above average deprivation and negative values below average deprivation. Zero values indicate areas where deprivation is at the Scottish average for that year.

The datasets include population weighted deciles and quintiles for all deprivation measures and single-variable indicators. To calculate deciles, Datazones were ordered by a deprivation measure and then split into 10 groups with 10% of all household population in each group. Quintiles were calculated by merging adjacent deciles, e.g. deciles 1 and 2 were merged into quintile 1. Higher quintile or decile values indicate higher deprivation. Table 3 shows the quintiles of the new deprivation measure. Since Datazones vary in population size, the number of Datazones in each quintile varies. The number of people should be roughly the same across quintiles and the percent of people in each quintile is the same when rounded to a single decimal. The table also shows average deprivation levels by quintiles.

The data also provides measures for self-reported general health and limiting long-term illness. For 2011 the data are for 5-year age groups (for both men and women), for 2001 the data are mostly for 10 and 15-year age groups. For both years we have calculated the standardized percentages for people in poor health and with limiting long-term health problems. For 2011 we were also able to provide standardized percentages for people in good health and with no limiting long-term illness. In all cases the standardization was done using the 2013 European Standard Population [9]. Since the census questions about health vary across the two censuses the percentages of people in ill health should not be compared across time. Please refer to the census metadata for census questions and their comparability across years [10].

The population breakdown is for the same age groups as the health data (for both men and women). The 2011 data will allow researchers to calculate further indices for health research, e.g. the slope and relative indices of inequality for self-reported health, similarly to what has been done by Allik et al. [1].

## Figures and Tables

**Table 1:** Data sources and table names, 2001.

Data	Source	Data table name
Social renting	Census	UV44.csv
NS-SeC of HRP and low social class of HRP	Commissioned from census	Not available otherwise
Qualifications	Commissioned from census	Not available otherwise
Unemployment	Census	CAS030.csv
General health and limiting long-term illness	Census	CAS016.csv
Male unemployment	Census	CAS028.csv
Overcrowding	Richardson [8]	GROS_2001_overcrowding_data.txt
Car ownership	Census	CAS022.csv
SIMD 2004	Scottish Government	0027000.csv
SG Urban Rural classification	Scottish Government	DZ2001_SE2003.txt

**Table 2:** Data sources and table names, 2011.

Data	Source	Data table name
Social renting	Census	QS403SC.csv
NS-SeC of HRP	Census	QS609SC.csv
Qualifications	Commissioned from census	CT_0033a_2011_DZ01.csv
Unemployment	Commissioned from census	CT_0033b_2011_DZ01.csv
General health	Commissioned from census	CT_0033d_2011_DZ01.csv
Limiting long-term illness	Commissioned from census	CT_0033e_2011_DZ01.csv
Carstairs score	Brown et al. [7]	Carstairs_scores_DZ2011.csv
SIMD 2012	Scottish Government	00410767.csv
SG Urban Rural classification	Scottish Government	DZ2001_SGUR2011_2012_Lookup.txt

**Table 3:** Quintiles of the new deprivation measure, 2011.

Quintile	1	2	3	4	5
Number of Datazones	1271	1253	1280	1334	1362
Number of people	1,039,179	1,039,216	1,038,950	1,039,102	1,039,939
Percent of people	20.0	20.0	20.0	20.00	20.0
Average deprivation score	−4.3	−2.6	−0.8	1.8	5.9
