# Supplementary material for: Small-area deprivation measure datasets for Scotland, 2001 and 2011
Source: Data Brief. 2016 Apr 30;7:1682–6. doi: 10.1016/j.dib.2016.04.060 (PMC5063812; doi:10.1016/j.dib.2016.04.060)
Supplement: Supplementary file 2 — Supplementary material [file mmc2.doc]

No conflict of interest.
